# Second Primary Malignancies and Their Associations With Age, Gender, and Latency Periods in Urogenital Cancers: A Retrospective Cohort Study

**DOI:** 10.7759/cureus.90017

**Published:** 2025-08-13

**Authors:** Faizan Fazal, Mohammad Ebad Ur Rehman, Arham A Khokhar, Saad Tahir, Usama Tanveer, Ali A Ijaz, Shahzaib Maqbool, Areesha Abid, Hafsa Arshad Azam Raja, Bilal Haider Malik

**Affiliations:** 1 Medicine, Holy Family Hospital, Rawalpindi, PAK; 2 Surgery, Oxford University Hospitals NHS Foundation Trust, Oxford, GBR; 3 Pediatric Surgery, Rawalpindi Medical University, Rawalpindi, PAK; 4 Surgery, Rawalpindi Medical University, Rawalpindi, PAK; 5 Dermatology, Betsi Cadwaladr University Health Board, Wrexham, GBR

**Keywords:** age group, latency interval, race, second primary malignancy, urogenital neoplasms

## Abstract

Objectives: This study aimed to identify the sites and risks of developing second primary malignancies (SPMs) after urogenital cancers, as well as their associations with race, gender, and age.

Methods: This was a retrospective cohort study involving 140,620 subjects with primary urogenital cancer. The data were extracted from the Surveillance, Epidemiology, and End Results (SEER) database, covering the duration from January 1975 to April 2025. This study included only those patients who were diagnosed with primary urogenital cancer. Descriptive statistics, absolute excess risk (AER), and observed-to-expected ratios (O/E) were calculated.

Results: Out of the 140,620 patients observed, 106,377 (75.64%) were male. Among the patients diagnosed with urogenital cancer, 33,680 (23.9%) developed an SPM. The mean age for the development of an SPM was 75.12 years. The risk of developing an SPM in patients with urogenital cancer was significantly higher compared to the general population (O/E = 1.31, confidence interval (CI) = 1.30-1.33, AER = 67.83). Male genital system cancers (O/E = 1.27, CI = 1.24-1.30, AER = 13.41), respiratory system cancers (O/E = 1.75, CI = 1.71-1.80, AER = 26.02), and urinary system cancers (O/E = 2.28, CI = 2.22-2.33, AER = 29.16) were among the most commonly identified SPMs. A total of 17,869 (53%) patients with an SPM belonged to the >75 years age group.

Conclusion: Compared to the general population, patients with urogenital cancer had a considerably higher risk of developing an SPM. Most SPM cases were observed in individuals aged 50-74 years and those over 75. A latency period of 12-59 months was commonly noted before the development of an SPM. The most frequently reported SPMs involved the male genital, respiratory, and urinary systems.

## Introduction

Urogenital cancers include all of the cancers of the urinary and genital tract, as well as germ cell tumours and tumours of external genitalia. They include some of the most common cancers worldwide, such as prostate cancer, as well as some of the rarest cancers, such as penile cancers, which occur in 12 per million male individuals in Europe [1,2]. Infectious agents such as* Chlamydia trachomatis *and *Mycoplasma genitalium *were found to be associated with an increased risk of ovarian cancers, while viruses such as hepatitis C and Epstein-Barr virus were found to be involved in renal neoplasms [3]. Arsenic, which is found in cigarette smoke, increases the risk of urothelial cancers [4]. Similarly, an association between obesity and an increased risk of aggressive prostate cancer was established in a study by Stewart et al. [5]. 

Biopsy remains the most important diagnostic tool, whereas cystoscopy and magnetic resonance imaging also continue to be used [6]. A single mode of treatment, such as surgery or radiation, may be used for early cancers, whereas multimodal therapy is usually preferred for advanced cancers, including chemotherapy with immune checkpoint inhibition therapy. However, the use may be limited to a subset of patients [6,7]. With increasingly better therapies available for urogenital cancers, the prognosis for these neoplasms has improved; moreover, this also increases the risk of the development of a second primary malignancy (SPM) [8]. This risk is particularly relevant in pediatric cases, where the long-term effects of treatment become more apparent over time [9]. The study aimed to investigate the elevated risk of SPMs following urogenital cancers and examine their associations with race, gender, and age. Additionally, it sought to identify and analyze the common sites of SPMs based on different age groups and genders.

A significantly increased frequency of sequential primary malignancy was seen in patients who were diagnosed with renal cell carcinoma in Norway [10]. One study identified prostate, lung, and bladder cancers as the most common SPMs following bladder cancer, while another found that after renal cell carcinoma, the most prevalent SPMs were prostate, colorectal, and bladder cancers in male patients and breast, colorectal, and lung cancers in female patients [11,12]. The answer to the question of what increased the risk of SPMs in prostate cancer was established to be radiotherapy [13]. 

The patients who developed lung cancer as the first primary malignancy, followed by an SPM, had a longer median survival than those who developed lung cancer as an SPM instead of as the first malignancy [14]. A smaller interlude of <1 year after diagnosis of renal cell carcinoma to diagnosis of an SPM was found to be the strongest predictor of poor overall survival (OS) in renal cell carcinoma patients with an SPM [15]. External beam radiotherapy (EBRT) and brachytherapy (BT) for prostate cancer were also found to statistically increase the incidence of both solid and hematopoietic second malignancies [16]. Recently, with improved treatments and better prognosis of cancers, the risk of SPMs, especially after radiotherapy, has been found to be increased due to long-term follow-up and increased OS. The occurrence of SPMs could be as high as 20% in patients who are treated with radiotherapy [17].

It is necessary to understand SPMs both for their treatments and to improve the quality of life of patients. A smaller population was investigated previously; however, we aimed to include a much larger population in our study. The development of SPMs after urogenital cancers shows an increased risk in comparison to the general population. We aimed to find out the increased risk of development of SPMs after urogenital cancers, as well as their associations with race, gender, and age. We also intended to identify and address the sites of SPMs in urogenital cancers in terms of age groups and genders.

## Materials and methods

Study design, subjects, and duration

This study was a retrospective cohort study involving 140,620 subjects with primary urogenital cancer. The study duration spanned from January 1975 to April 2025.

Data source

The data were extracted from the Surveillance, Epidemiology, and End Results (SEER) database, which is a data registry funded by the National Cancer Institute. SEER is the largest publicly available cancer patient database. It monitors the annual incidence of cancer diagnoses and gathers follow-up data on all patients who have received a cancer diagnosis until the time of death.

Eligibility criteria

Eligibility criteria were clearly defined before the initiation of the study. This study included only those patients who were diagnosed with primary urogenital cancer, confirmed by histopathological reports in the database. We excluded all patients with any other type of primary malignancy, those who had no follow-up, or those for whom urogenital cancer wasn’t the primary cancer. Patients with incomplete details on the variables being studied were also excluded. The eligibility criteria were strictly set to include only those patients with primary urogenital cancer and with complete details.

Outcome variables and statistical analysis

We collected information about gender (male and female), age (0-49, 50-74, +75), race (White, Black, Other), metastasis to different anatomical sites (gastrointestinal system, liver, respiratory system, skin, genitourinary system), latency (the period from the diagnosis of primary urogenital cancer to the diagnosis of a second malignancy), odds ratio (OR), and adjusted hazard ratio (HR). Demographic characteristics (gender, age, and race) were analyzed using descriptive statistics, and percentages were calculated. The observed-to-expected ratio (O/E) was calculated by dividing the number of observed cases of SPMs in urogenital cancers by the expected cases of SPMs. Absolute excess risk (AER) was calculated by subtracting the risk for the non-exposed group from the risk for the exposed group.

## Results

Demographic details of the study population

The number of urogenital cancer patients observed in our retrospective cohort was 140,620. Out of these, 33,680 (23.9%) developed a second malignancy. The mean age for the development of an SPM was 75.12 years. Among all patients observed, 106,377 (75.64%) were male and 34,243 (24.35%) were female. These demographic details are provided in Table 1.

**Table 1:** Demographic details of SPM patients with urogenital cancer 'Other' races include American Indian/Alaska Native, Asian/Pacific Islander. SPM: Second primary malignancy

Characteristics	Frequency, n (%)
Total number of patients who developed SPMs	33,680 (23.9%)
Gender	
Male	106,377 (75.64%)
Female	34,243 (24.35%)
Race	
White	128,699 (91.5%)
Black	4,840 (3.4%)
Other	7,081 (5.1%)

SPM sites in urogenital cancer patients

The risk for developing an SPM in patients with urogenital cancer was significantly higher compared to the general population, with an O/E of 1.31, a CI of (1.3-1.33), and an AER of 67.83. Male genital system cancers (N = 7,555, O/E = 1.27, CI = 1.24-1.3, AER = 13.41), specifically prostate cancers (N = 7,483, O/E = 1.27, CI = 1.24-1.3, AER = 13.34), respiratory system cancers (N = 7,180, O/E = 1.75, CI = 1.71-1.8, AER = 26.02), and urinary system cancers (N = 6,172, O/E = 2.28, CI = 2.22-2.33, AER = 29.16), specifically urinary bladder cancers (N = 3,682, O/E = 1.92, CI = 1.86-1.99, AER = 14.9), were among the most common SPMs identified in our study, as shown in Table 2. We also identified an increased risk of SPMs in areas such as the liver, gallbladder, intrahepatic bile ducts, and other biliary organs, skin, kidney, ureter, and renal pelvis, as shown in Table 2.

**Table 2:** SPM sites in urogenital cancer patients NOS: Not otherwise specified; O/E: Observed-to-expected ratio; AER: Absolute excess risk; SPM: Second primary malignancy

SPM	Observed	O/E	Confidence interval	AER
All sites	33,680	1.31	1.3-1.33	67.83
All sites excluding non-melanoma skin	33,541	1.32	1.3-1.33	67.8
Digestive system	5,384	1.03	1-1.06	1.31
Small intestine	143	1.38	1.16-1.62	0.33
Large intestine, NOS	111	0.82	0.68-0.99	-0.02
Liver, gallbladder, intrahepatic bile duct, and other biliary organs	576	1.09	1-1.8	0.41
Intrahepatic bile duct	77	1.37	1.08-1.72	0.18
Respiratory system	7,180	1.75	1.71-1.8	26.02
Larynx	319	1.35	1.2-1.5	0.69
Lung, bronchus, trachea, mediastinum, and other respiratory organs	6,815	1.79	1.74-1.83	25.26
Skin excluding basal and squamous	1,045	0.84	0.79-0.9	-1.63
Melanoma of the skin	906	0.82	0.77-0.88	-1.66
Corpus and uterus, NOS	218	0.87	0.76-1	-0.27
Corpus uteri	212	0.87	0.76-1	-0.26
Vagina	22	2.58	1.62-3.91	0.11
Vulva	46	1.42	1.04-1.89	0.11
Male genital system	7,555	1.27	1.24-1.3	13.41
Prostate	7,483	1.27	1.24-1.3	13.34
Testis	28	1.68	1.11-2.43	0.1
Urinary system	6,172	2.28	2.22-2.33	29.16
Urinary bladder	3,682	1.92	1.86-1.99	14.9
Kidney and renal pelvis	1,517	2.11	2.01-2.22	6.73
Renal pelvis, ureter, and other urinary organs	1,626	10.66	10.15-11.9	12.42
Kidney	864	1.34	1.25-1.43	1.84
Ureter	651	13.17	12.8-14.22	5.07
Other urinary organs	322	10.57	9.45-11.79	2.46
Lymphocytic leukemia	373	0.89	0.8-0.98	-0.39
Chronic lymphocytic leukemia	325	0.87	0.78-0.97	-0.42
Non-lymphocytic leukemia	457	1.12	1.02-1.23	0.42
Acute non-lymphocytic leukemia	309	1.19	1.06-1.34	0.42
Myeloid and monocytic leukemia	404	1.16	1.05-1.28	0.47
Acute myeloid leukemia	251	1.14	1.01-1.29	0.27
Acute monocytic leukemia	23	1.89	1.2-2.84	0.09
Aleukemic, sub-leukemic, and NOS	18	0.56	0.33-0.89	-0.12
Kaposi sarcoma	10	0.48	0.23-0.89	-0.09
Miscellaneous	1,127	1.10	1.03-1.16	0.84

Correlation of age with SPMs

Regarding the correlation of age with SPMs, out of 33,680 patients in whom SPMs were observed, 330 (0.9%) patients belonged to the 0-49 years age group (O/E = 2.75, CI = 2.46-3.06, AER = 38.63), 15,481 (45.9%) patients belonged to the 50-74 years age group (O/E = 1.48, CI = 1.46-1.5, AER = 82.9), and 17,869 (53.2%) patients belonged to the >75 years age group (O/E = 1.19, CI = 1.17-1.21, AER = 53.58). These details are shown in Table 3.

**Table 3:** Correlation of age with SPMs O/E: Observed-to-expected ratio; AER: Absolute excess risk; SPM: Second primary malignancy

Age groups	Patients with SPM, n (%)	O/E	Confidence interval	AER
0-49 years	330 (0.9%)	2.75	2.46-3.06	38.63
50-74 years	15,481 (45.9%)	1.48	1.46-1.5	82.9
>75 years	17,869 (53.2%)	1.19	1.17-1.21	53.58

Relationship between SPMs in different organs and age groups

In 330 SPM patients aged 0-49 years, the risk of malignancy in the ureter (O/E = 340.70, CI = 186.27-571.64, AER = 2.57), renal pelvis (O/E = 91.96, CI = 47.51-160.63, AER = 2.18), and urinary bladder (O/E = 15.70, CI = 11.89-20.34, AER = 9.81) was significantly higher compared to the general population. Furthermore, SPMs were observed in greater numbers at sites such as the prostate (O/E = 13.17, CI = 10.41-16.44, AER = 13.25) and breast (N = 27, O/E = 1.45, CI = 0.96-2.11, AER = 1.55).

In 15,481 SPM patients aged 50-74 years, the risk of malignancy in the ureter (O/E = 21.33, CI = 19.11-23.75, AER = 5.27), renal pelvis (O/E = 13.88, CI = 12.4-15.48, AER = 4.95), and vagina (O/E = 3.44, CI = 1.57-6.52, AER = 0.11) was significantly higher compared to the general population. Additionally, SPMs were observed in greater numbers at sites such as the prostate (O/E = 1.46, CI = 1.42-1.51, AER = 22.17) and lung and bronchus (O/E = 1.93, CI = 1.86-2, AER = 24).

In 17,869 SPM patients aged >75 years, the risk of malignancy in the ureter (O/E = 8.98, CI = 8-10.06, AER = 5.1) and renal pelvis (O/E = 6.47, CI = 5.78-7.22, AER = 5.11) was significantly higher compared to the general population. Additionally, SPMs were observed in greater numbers at sites such as the lung and bronchus (O/E = 1.68, CI = 1.63-1.74, AER = 28.98), prostate (O/E = 1.06, CI = 1.02-1.09, AER = 3.24), and urinary bladder (O/E = 1.58, CI = 1.51-1.65, AER = 14.25). This is shown in Table 4.

**Table 4:** Relationship between SPMs in different organs and age groups O/E: Observed-to-expected ratio; AER: Absolute excess risk; SPM: Second primary malignancy

SPM in organs	Patients with SPM (n)	O/E	Confidence interval	AER
0-49 years age group				
Ureter	14	340.70	186.27-571.64	2.57
Renal pelvis	12	91.96	7.51-160.63	2.18
Urinary bladder	57	15.70	11.89-20.34	9.81
Prostate	78	13.17	10.41-16.44	13.25
Breast	27	1.45	0.96-2.11	1.55
50-74 years age group				
Ureter	334	21.33	19.11-23.75	5.27
Renal pelvis	322	13.88	12.4-15.48	4.95
Vagina	9	3.44	1.57-6.52	0.11
Prostate	4,248	1.46	1.42-1.51	22.17
Lung and bronchus	3,009	1.93	1.86-2	24
Colon, rectum, and anus	1,078	1.03	0.97-1.09	0.46
>75 years age group				
Ureter	303	8.98	8-10.06	5.1
Renal pelvis	319	6.47	5.78-7.22	5.11
Lung and bronchus	3,773	1.68	1.63-1.74	28.98
Prostate	3,157	1.06	1.02-1.09	3.24
Urinary bladder	2,048	1.58	1.51-1.65	14.25

Occurrence of SPMs during different latency periods

Regarding the latency period, out of 33,680 urogenital cancer patients with SPMs, 5,473 (16.2%) patients developed an SPM within 2-11 months. The risk of developing an SPM in the ureter (O/E = 23.05, CI = 18.62-28.2, AER = 8.31) and renal pelvis (O/E = 20.38, CI = 16.91-24.35, AER = 10.63) was quite high compared to the general population. Within this latency period, prostate malignancies (O/E = 4.63, CI = 4.45-4.82, AER = 172.52) and urinary bladder malignancies (O/E = 5.43, CI = 5.06-5.81, AER = 62.35) were the most common malignancies.

Furthermore, 11,015 (32.7%) patients developed an SPM within 12-59 months. The risk of developing an SPM in the ureter (O/E = 12.00, CI = 10.32-13.87, AER = 4.28) and renal pelvis (O/E = 9.87, CI = 8.61-11.27, AER = 5.03) was quite high compared to the general population. Within this latency period, lung and bronchus malignancies (O/E = 1.87, CI = 1.79-1.95, AER = 26.86) and prostate cancers (O/E = 1.20, CI = 1.15-1.25, AER=9.74) were the most common malignancies. The rest of the details are provided in Table 5.

**Table 5:** Occurrence of SPMs during different latency periods O/E: Observed-to-expected ratio; AER: Absolute excess risk; SPM: Second primary malignancy

Organs	Patients with SPM (n)	O/E	Confidence interval	AER
2-11 months latency period				
Ureter	94	23.05	18.62-28.2	8.31
Renal pelvis	121	20.38	16.91-24.35	10.63
Prostate	2,381	4.63	4.45-4.82	172.52
Urinary bladder	827	5.43	5.06-5.81	62.35
12-59 months latency period				
Ureter	182	12.00	10.32-13.87	4.28
Renal pelvis	218	9.87	8.61-11.27	5.03
Lung and bronchus	2,252	1.87	1.79-1.95	26.86
Prostate	2,300	1.20	1.15-1.25	9.74
60-119 months latency period				
Ureter	207	16.18	14.05-18.54	6.27
Renal pelvis	161	8.63	7.35-10.07	4.59
Lung and bronchus	1,768	1.77	1.68-1.85	24.74
Prostate	1,397	0.88	0.83-0.92	-6.39
>120 months latency period				
Ureter	168	9.67	8.26-11.25	3.98
Renal pelvis	153	5.90	5-6.91	3.35
Lung and bronchus	2,228	1.74	1.67-1.81	24.98
Prostate	1,405	0.75	0.71-0.79	-12.3

Correlation of race with SPMs

In terms of the comparison of different races, out of 33,680 urogenital cancer patients with SPMs, 31,332 patients were of the White race, 941 patients were of the Black race, and 1,407 patients belonged to 'Other' races (American Indian/Alaska Native, Asian/Pacific Islander). Among the White race, there was a risk of developing SPMs in the renal pelvis, ureter, and other urinary organs (N = 1,511, O/E = 10.41, CI = 9.9-10.95, AER = 12.42).

## Discussion

The term SPM is used to describe malignancies that are related to prior cancers but arise independently, i.e., not as a result of recurrence or metastasis [18]. New healthcare technologies and treatments have improved the survival rate of cancer patients. However, this prolonged survival has also been accompanied by an increase in the prevalence of SPMs [19]. Prolonged survival allows the carcinogenic process to occur, and older tissues are more susceptible to carcinogenesis. This, coupled with the adverse effects of radiotherapy and chemotherapy across different genetic backgrounds, is considered a risk factor for the development of SPMs [20]. As a result, SPMs have become a source of growing concern among healthcare professionals. 

In this study, we aimed to determine the risk of developing SPMs after urogenital cancers and the possible role of other factors such as gender, race, and age. Previous studies have shown that bladder cancer, in particular, is associated with a high risk of developing SPMs [21]. Similarly, in our study, we found that patients with urogenital cancer had a significantly increased risk of developing SPMs as compared to the general population. SPMs most commonly occurred in the urogenital and respiratory systems. A possible cause of this may be smoking, as it is a risk factor for the development of both urogenital cancers and respiratory system cancers [22,23]. In our study, we found that most patients who developed SPMs were male. This is consistent with prior studies regarding SPM incidence with other types of cancers [24]. Many factors may explain this observation, such as lifestyle differences, with male individuals being more exposed to carcinogens due to occupational settings. Genetic predisposition has also been implicated [25].

Regarding race, White individuals were found to be significantly more susceptible to developing SPMs compared to other races. Other studies have also identified differences in the risk of developing SPMs among various racial groups; however, the causes of these differences remain unclear [26,27]. Genetic predisposition and lifestyle differences have been implicated as plausible factors [28].

Most patients with SPMs in our study belonged to the older age group. This comes as no surprise, as the increased risk of cancer with advancing age is well-documented. Older tissues are more susceptible to carcinogenesis due to changes in the cellular microenvironment associated with aging [29]. We observed that patients with urogenital cancer had a considerably higher risk of developing SPMs in comparison to the general population. This is consistent with similar studies conducted elsewhere in the world. Adult bladder cancer patients have a much higher likelihood of developing SPMs compared to the general population. Survivors of bladder cancer may benefit from a lifetime of monitoring for the occurrence of SPMs [11]. Our study also confirmed that the occurrence of SPMs is more common among older individuals. Although SPMs are becoming more common, the prognosis is not promising. Both the prevention and treatment of SPMs require greater focus. Most of the patients were older than 65 years of age [19]. Thus, studies suggest that the incidence of SPMs increases with age, a finding we also observed in our study.

The findings of this study hold great importance in enhancing our knowledge base of SPMs associated with urogenital cancers. Due to the large population size, this study provides greater reliability than previously conducted studies. It is hoped that with these findings, healthcare professionals will be better equipped to care for cancer patients. As SPMs pose a growing challenge, it is essential for doctors to be aware of various implicated risk factors so that early recognition and treatment are possible.

The limitation of this study is that it recorded the metastasis of primary urogenital cancer to different sites only in patients residing in the United States. Publication bias and missing data may have influenced the reported outcomes; therefore, the results may not apply to the global population. Nevertheless, it is a one-of-a-kind study and offers deep insights into the various dynamics related to SPMs observed in patients with urogenital cancers.

## Conclusions

The majority of SPM cases were observed in male patients. Approximately one-fourth of the patients with urogenital cancers developed an SPM. In comparison to the general population, patients with urogenital cancer had a considerably higher risk of developing an SPM. The vast majority of SPM cases belonged to the age groups of 50-74 years and >75 years. A latency period of 12-59 months was a relatively prevalent time frame observed for the development of SPMs in these patients. Cancers of the male genital system, respiratory system, and urinary system were among the most common SPMs identified in our study.
